# Suitability of Selected Apple Varieties for People with Allergies and Diabetes

**DOI:** 10.3390/nu16132109

**Published:** 2024-07-02

**Authors:** Mateusz Aninowski, Joanna Leszczyńska, Radosław Bonikowski, Alicja Ponder, Ewelina Hallmann, Małgorzata Grzyb, Kamil Szymczak

**Affiliations:** 1 Department of Pathophysiology, Institute of General and Experimental Pathology, Medical University of Lodz, Zeligowskiego 7/9, 90-752 Lodz, Poland; mateusz.aninowski@umed.lodz.pl; 2 Institute of Natural Products and Cosmetics, Faculty of Biotechnology and Food Sciences, Lodz University of Technology, Stefanowskiego 2/22, 90-537 Lodz, Poland; radoslaw.bonikowski@p.lodz.pl (R.B.); malgorzata.grzyb@p.lodz.pl (M.G.); 3 Department of Functional and Organic Food, Institute of Human Nutrition Sciences, Warsaw University of Life Sciences, Nowoursynowska 159c, 02-776 Warsaw, Poland; alicja_ponder1@sggw.edu.pl (A.P.); ewelina_hallmann@sggw.edu.pl (E.H.); 4 Bioeconomy Research Institute, Agriculture Academy, Vytautas Magnus University, Donelaicio 58, 44248 Kaunas, Lithuania

**Keywords:** apples, polyphenols, allergenic proteins, allergy, diabetes

## Abstract

The study aimed to select apple varieties suitable for allergy sufferers and people with diabetes. The total polyphenol content, sugar content, acidity, and antioxidant activity of the apple fruit juices were determined using spectrophotometric techniques. The allergenic content in the apple juices was also measured. The strength of sensitisation was assessed using the ELISA method. Given their minimal content of both profilins and Bet v 1 homologues, Koksa Pomarańczowa (4.24 ± 0.08 µg/g Bet v 1 and 4.49 ± 0.82 ng/g profilins) and Książę Albrecht Pruski (5.57 ± 0.07 µg/g Bet v 1 and 3.34 ± 0.09 ng/g profilins) were identified as suitable for people with allergies. For people with diabetes, the most suitable apple variety was found to be Jakub Lebel, providing large doses of antioxidants and polyphenols (41.10 ± 0.20 and 5.16 ± 0.42, respectively) and a relatively low sugar content (9.06 g/100 g).

## 1. Introduction

It has long been known that unhealthy diets contribute to the incidence of non-communicable so-called “diseases of civilisation”, including diabetes. Diabetes is reported to affect over 450 million people worldwide, and this figure is expected to increase to 700 million by 2045. The most common type 2 diabetes is a global health problem characterised by a set of metabolic disorders, including insulin resistance, hyperglycemia, and dyslipidemia, with possible complications in the form of non-alcoholic fatty liver disease (NAFLD), central obesity, cardiovascular diseases, and hypertension [1]. These diseases are often significant contributors to mortality and morbidity [2]. In response, present-day population and individual strategies are focused on risk prevention and developing personalised dietary solutions. This approach is based on the understanding that modifiable lifestyle factors, particularly diet and nutrient intake, play a significant role in diseases’ aetiology and progression [3]. It also assumes that universal dietary recommendations will work similarly for all individuals within different populations. However, this approach often ignores differences between individuals regarding dietary needs. Considering these differences opens the possibility of achieving better health outcomes in the general population [4,5,6].

The apple (*Malus domestica* L. Borkh), belonging to the rose family (Rosaceae), is cultivated and consumed all over the world [7]. Apple tree fruits are a rich source of nutrients and nutraceuticals and are recommended components of a healthy diet. Eating apples can also reduce the risk of cardiovascular diseases and type 2 diabetes [8]. Apples are one of the fruits with the highest polyphenol content [9]. Both the peel and the pulp are rich in polyphenols, including catechins, quercetin, rutin, phlorizin, phloretin, and chlorogenic acid, which have positive effects on health, especially by limiting the development of neurodegenerative diseases. In vitro studies on mice have shown that polyphenols extracted from apples can protect gastrointestinal mucosa damaged by drugs [9]. Various other in vitro studies and clinical trials have demonstrated that polyphenol-rich foods such as apples can positively affect human and animal health by stimulating the immune system [10]. In particular, the outer layer of the apple contains enzymatic and non-enzymatic antioxidants, which are valuable for maintaining homeostasis [10]. Polyphenols are known for their potential to reduce the allergenicity of various food sources, including apples [11]. However, despite their high polyphenol content, apples can cause allergies [12]. Allergies to fruits like apples are predominantly associated with pollinosis.

In North and Central Europe, sensitisation to apples is caused mainly by cross-reactive birch pollen aeroallergens. Allergic reactions to apple fruit are also exhibited by people allergic to birch pollen. That is due to the cross-reaction between the main birch pollen allergen Bet v1 and its structural homologue Mal d 1, which is the main apple allergen. Both belong to the pathogenesis-related protein 10 (PR-10) family, indicating that they play an essential role in plant defence. Mal d 1, a heat-labile protein sensitive to pepsin digestion, causes relatively mild and local symptoms, including itching, tingling, or swelling of the lips, tongue, and throat, known as oral allergy syndrome (OAS) [13]. In South Europe, apple allergy is not associated with birch pollen allergy. Instead, it is associated with peach allergies, which have more severe symptoms. This type of allergy may be related to the non-specific lipid transfer protein (nsLTP) Mal d 3, which is high temperature and enzymatic digestion resistant. Other apple allergens include profilin Mal d 4 and thaumatin-like protein Mal d 2 [14,15]. The allergenicity of apples differs across cultivars [12].

In this study, different apple varieties were examined regarding their health-promoting properties, sensory characteristics, and allergen content. Identifying apple varieties with reduced allergenicity and low sugar content could help people with diabetes and allergy sufferers. The results could help to inform patients with allergies or diabetes dietary recommendations, as it would be possible to distinguish food products with lower or higher sugar content and allergenicity.

## 2. Materials and Methods

### 2.1. Fruit Material and Sampling Design

For this study, the following 30 apple varieties were selected: Boskoop, Deans’ Codlin, Galloway Pippin, Grafsztynek Inflancki, Grochówka, Jakub Lebel, James Grieve, Kalwila Aderslebeńska, Kantówka Gdańska, Koksa Pomarańczowa, Kosztela, Kronselska, Krótkonóżka Królewska, Książę Albert, Książę Albrecht Pruski, Malinowa Oberlandzka, Niezrównane Peasgooda, Pepina Linneusza, Pepina Ribstona, Piękna z Rept, Reneta Blenheimska, Reneta Herberta, Reneta Kanadyjska, Reneta Kulona, Reneta Strauwalda, Reneta z Brownlee, Schieblers Taubenapfel, Szara Reneta, Złota Reneta, and Złotka Kwidzyńska. The fruit samples were sourced from the Institute of Horticulture in Skierniewice, home to one of Poland’s most extensive collections of cultivated apple varieties. These selections were gathered during 2018–2019, and all varieties originated from the same cultivation site [16]. For each variety, there were 5 fruit samples chosen for further studies.

After being transported to the chemical laboratory, the fresh fruits were gently washed, and the green stems were cut off. Each fruit was cut in half to remove the stalk, seeds, seed shells, seed chamber, calyx, and the calyx recess. Then, 3 g of the tissue was placed in a bowl, and the fruit was crushed with a plunger to extract the juice without fruit peel. After extraction, 100 µL of juice was placed into Eppendorf tubes, and 900 µL of deionised water was added. The solutions were then directed for further analysis. Sensory evaluations were conducted on cubed samples of the fresh fruit. For acidity analysis, the fruits were dried (peel and pulp separately). The drying process was carried out using a temperature-controlled fruit dryer. Before drying, the pulp was cut into thin slices to ensure the same drying time for both pulp and peel. The drying process was carried out at 60 °C for 24 h. After this time, no weight loss was observed in the samples. Prepared samples were stored at 4 °C.

### 2.2. Analysis of Potential Allergenicity

The following reagents were used: 3% solution of skimmed milk powder (Piątnica, Warsaw, Poland); PBS (phosphate buffered saline); Tween 20 (Sigma-Aldrich, St. Louis, MO, USA); 3 M NaOH solution (Sigma-Aldrich, USA); mouse antibodies against Bet v I (Dendritics, France); anti-mouse antibodies (conjugate with the phosphatase enzyme, produced in goat by Sigma-Aldrich, St. Quentin Fallavier, France); and PNPP solution (*p*-nitrophenol phosphate ready solution, produced by Sigma Life Science, Wroclaw, Poland). The main pollen allergen was Profilin-1 Bet v II (H CUSABIO, Houston, TX, USA).

The potentially allergenic protein homologous to Mal d 1 was determined using a cross-reaction with an antibody against Bet v 1. Analyses were performed according to previously described procedures [7]. Each well in 96-well polystyrene microplates (Medium F96 by Nunc, DK) was filled with 100 μL of standard solution, and a standard curve containing Bet v 1 concentrations of [µg/g] 0.5, 2.5, 5, and 25, profilin concentrations of [ng/g] 0.5, 5, 10, and 50, as well as samples from extracts, were diluted three times. The plate was placed in a refrigerator (temperature of 4 °C) for 12 h. After this time, 4 × 350 μL of Tween’s PBS wash buffer was added to each used well. Then, the plate was incubated for 2 h after adding 400 μL of 3% skimmed milk solution to the wells, forming a convex meniscus. The wells were washed using 4 × 350 μL PBS buffer with Tween. Then, 100 μL of mouse antibodies against Bet v 1 (diluted 1000 times) were added and incubated for 1 h at room temperature. Then, the plate was washed with 4 × 350 μL wash buffer. Goat antibodies against mouse immunoglobulins (100 μL) conjugated with alkaline phosphatase (diluted 5000 times) were added to each well and incubated for 1 h. After this time, 4 × 350 μL of washing buffer were washed, and 100 μL of substrate for the pNPP enzyme were added. After 30 min, a blue product was formed. The reaction was stopped by adding 100 μL of a 3 M NaOH stop solution. The absorbance was read at a wavelength of 405 nm using a Multiskan RC microplate test reader with computer software—Genesis program (ver. 5.0), ThermoLabsystem FL (Waltham, MA, USA). The allergen content was calculated according to the appropriate standard curve equation.

Profilin analogues were also identified. The test was performed analogously to the indirect ELISA test to determine Bet v 1 analogues, but using antibodies that detect profilins.

### 2.3. Sensory Assessment

Before the sensory evaluation of the apple samples, the test subjects were familiarised with the general taste assessment scale for sweetness and sourness. The test subjects were trained in sensory assessment. Experts of different genders were asked to lightly rinse their mouths with each solution, spit it out, and then drink a glass of water. After waiting 15 min, they received apple samples for evaluation.

After warming to room temperature, the apples were washed and wiped dry, then cut into eights with the core removed. Immediately after preparation, they were given to experts for evaluation. The experiment was conducted using a single-blind trial method. The experts were asked to give each assessed feature a value from 0 to 9. They received four solutions for comparison:

1. Apple juice diluted twice with still mineral water (sweetness and sourness close to 0);
2. Apple juice diluted twice with still mineral water with the addition of malic acid to obtain a concentration of 10 g/L (sweetness close to 0, sourness up to 9);
3. Apple juice diluted twice with still mineral water with the addition of fructose to obtain a concentration of 60 g/L and sucrose to obtain a concentration of 60 g/L (acidity close to 0, sweetness up to 9);
4. Apple juice diluted twice with still mineral water with the addition of fructose to obtain a concentration of 60 g/L and sucrose to obtain a concentration of 60 g/L and with the addition of malic acid to obtain a concentration of 10 g/L (sweetness and sourness close to 9).

Then, the experts were asked to lightly rinse their mouths with each solution, spit it out, and drink a glass of water. After waiting 15 min, they received the next apple sample for evaluation. Each variety was assessed by a minimum of 6 and a maximum of 9 experts.

### 2.4. Determination of Acidity

The following reagents were used: 0.1 M aqueous NaOH solution p.a. (Chempur, Piekary Śląskie, Poland). The apple acidity was determined using the PN-EN 12147:2000 standard [17]. The samples were washed under running water and wiped dry, then cut into small pieces (with the peel). Next, 5 g of fruit was ground in a mortar. After fine grinding, 2 g of the sample was transferred to a beaker, 25 mL of distilled water was added, and titration was performed with a 0.1 M aqueous NaOH solution. An automatic titrator was used (SI Analytics TitroLine Easy, Mainz, Germany). Based on a previous study [16], the endpoint of the titration was a pH value equal to 8.1. The analyses were repeated twice or three times for major differences between values.

### 2.5. Determination of Sugars

The following reagents were used: 99.8% GC methanol (Sigma-Aldrich, Poznań, Poland); 99% pyridine (Sigma-Aldrich, Poznań, Poland); and N,O-Bis(trimethylsilyl)trifluoroacetamide with trimethylchlorosilane, BSTFA + TMCS (99:1) (Sigma-Aldrich, St. Quentin Fallavier Cedex, France). The dried pulp was ground in a mortar into a fine powder. Then, 0.5 g samples were placed in extraction thimbles in Soxhlet apparatuses. The samples were extracted using 60 mL of methanol (approx. 50 mL extraction chamber volume). The samples were then evaporated to dryness on a rotary evaporator. The flasks were left open until the next day to evaporate the remaining solvent. Subsequently, 2 mL of methanol was added and dissolved using an ultrasonic bath until a homogeneous sample was obtained. The extracts were quantitatively transferred to 2 mL glass tightly capped vials. For analysis, 20 μL of each extract was added to chromatographic vials and evaporated to dryness under a gentle stream of nitrogen. Then, 100 µL of pyridine and 100 µL of BSTFA + TMCS (99:1) were added. The vials were tightly capped and placed on a heating plate at 60 °C for 30 min. After this time, the samples were transferred to microinserts and placed on the tray of a Gerstel MultiPurpose Sampler (MPS 2). GC-MS analyses were performed on a Pegasus 4D device by LECO, equipped with an Agilent 7890A (Santa Clara, CA, USA) gas chromatograph coupled with a time-of-flight mass spectrometer. The compounds were separated on a BPX5 column (30 m × 0.25 mm × 0.25 µm) from SGE (Trajan Scientific, Ringwood, VIC, Australia). The carrier gas was helium at a flow of 1.5 mL/min. The temperature program began at 50 °C for 2 min, followed by an increase of 5 °C/min to 300 °C and held for 1 min (total analysis time was 53 min). The oven temperature of the second dimension column was 5 °C higher than that of the first column. The dispenser temperature was 250 °C. The mass spectrometer was operated in the electron impact mode at −70 eV and in the scanning range (*m*/*z*) from 29 to 950 amu. The ion source temperature was 200 °C, and the transfer line was 250 °C.

### 2.6. Determination of Antioxidant Potential

The following reagents were used: 99.8% GC methanol (GC, Sigma-Aldrich, Poland); 2,2-diphenyl-1-picrylhydrazyl radical, DPPH∙ (Sigma-Aldrich, Poland); 97% Trolox (Sigma-Aldrich, France). The potential antioxidant was determined using the DPPH and FRAP methods. Quantitative analyses were based on a standard curve using Trolox at the following concentrations: 5 mM, 2.5 mM, 1 mM, 0.5 mM, 0.25 mM, and 0.1 mM. In the DPPH method, 2 µL of the tested extract was transferred to a well on a 96-well plate, and 250 µL of DPPH∙ reagent was added. Three replicates were performed for each sample. After preparing the samples, the plate was covered to protect against oxygen access and incubated in a dark place for 30 min. After this time, absorbance was measured at a wavelength of 517 nm in a Multiscan GO device (ThermoFisher Scientific, Waltham, MA, USA). The results were converted from the standard curve to an equivalent amount of Trolox (mg TE/g). In the FRAP method, 290 μL of a solution prepared from acetate buffer with a pH equal to 3.6, 10 mM TPTZ solution, and 20 mM FeCl_3_⋅6H_2_O solution (mixed in a volume ratio of 10:1:1) were transferred to a 96-well, to which 10 μL of the extract of the tested sample were added and mixed. After 8 min, the absorbance was measured at λ = 593 nm using a Multiscan GO instrument (ThermoFisher Scientific, USA).

### 2.7. Determination of Total Polyphenol Content

The following reagents were used: Folin–Ciocalteu reagent (Chempur, Poland); 99.8% gallic acid (Sigma-Aldrich, Poland); sodium carbonate, Na_2_CO_3_, part d.a. (Chempur, Poland); 99.8% GC methanol (Sigma-Aldrich, Poland). The total polyphenol content was determined using the Folin–Ciocalteu reagent method. Polyphenolic compounds were selected based on the literature, including the most commonly used gallic acid [18]. For each series of determinations, a standard curve was prepared using gallic acid at concentrations of 25 mg/L, 50 mg/L, 75 mg/L, 100 mg/L, 200 mg/L, 300 mg/L, and 400 mg/L in methanol. To calculate the total polyphenol content of the extracts, 2 µL of the tested extract was added to a well on a 96-well plate, to which 4 µL of Folin–Ciocalteu’s reagent, 40 µL of 20% Na_2_CO_3_, and 250 µL of distilled water were added. Three replicates were performed for each sample. After all samples were prepared, the plate was placed in a dark place for 30 min. After this time, absorbance was measured at a wavelength of 750 nm. After scanning the plates using a Multiscan GO camera, the results were converted using the standard curve to the equivalent amount of gallic acid (mg GAE/100 g).

### 2.8. Statistical Analysis

All results were statistically tested with a one-way analysis of variance and a post hoc Tukey HSD test with a significance level of *p* < 0.05. Statistical analysis was performed using Statistica Version 10 (StatSoft, Tulsa, OK, USA). The Pearson’s r correlation coefficient was also used to develop the research results.

## 3. Results

### 3.1. Allergenicity Results

The highest content of Bet v 1 homologues was found in the varieties Złotka Kwidzyńska (12.40 µg/g), Malinowa Oberlandzka (9.86 µg/g), and Jakub Lebel (8.87 µg/g). The varieties with the highest profilin content were Reneta Blenheimska (8.81 ng/g), Szara Reneta (7.88 ng/g), Pepina Ribstona (7.67 ng/g), Szara Reneta (7.64 ng/g), and Reneta z Brownlee (7.63 ng/g). A clear correlation was observed between the contents of individual allergens in each variety. The Pearson’s r correlation coefficient for the two sets was 0.55, suggesting a clear positive correlation (the higher the Bet v 1 homologues content, the higher the profilin content). The closer to 1, the stronger the interaction between the results (Table 1). Kosztela and Kantówka Gdańska had the lowest content of Bet v 1 homologues (4.21 and 4.68 µg/g, respectively). Reneta Harberta and Schieblers Taubenapfel had the lowest content of profilins (1.74 and 2.26 ng/g, respectively) (Table 1). These varieties may be better tolerated by people suffering from cross-allergies, especially those caused by profilins, which combine pollinosis and food allergies.

**Figure 1:**
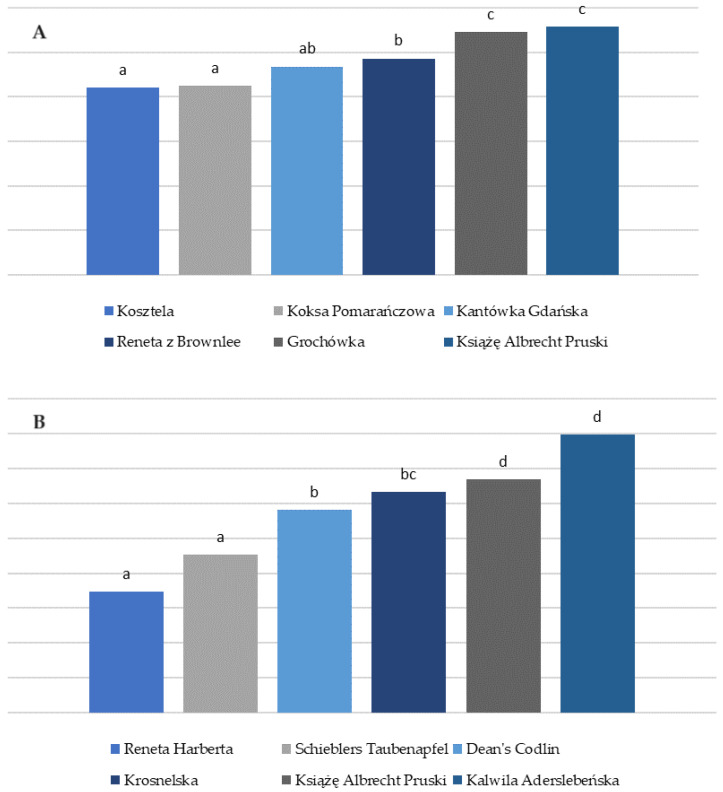
Apple varieties characterised by the lowest content of (**A**) Bet v 1 homologs (**B**) profilins. Different letters indicate statistical differences between samples at *p* < 0.05.

Analysing the above tables and ranking the varieties characterised by the lowest content of Bet v I homologs and profilins are gathered in Figure 1.

### 3.2. Sensory Assessment

The apple varieties were subjected to sensory analysis. The average results are presented in Table 2.

**Figure 2:**
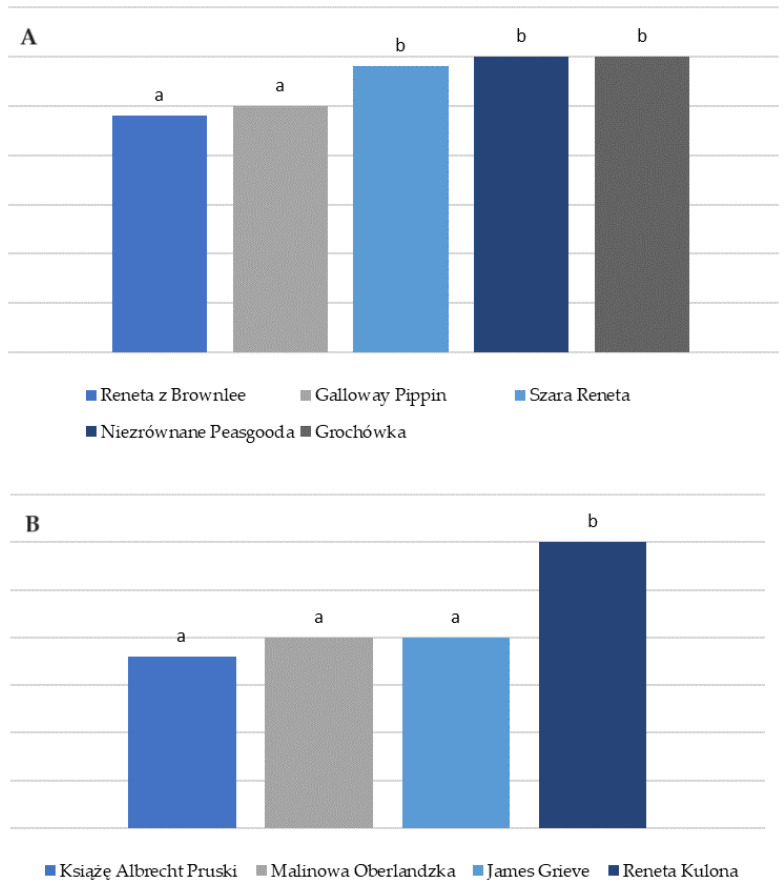
Apple varieties characterised by the lowest of (**A**) sweetness and (**B**) sourness. Different letters indicate statistical differences between samples at *p* < 0.05.

According to the experts in the sensory evaluation tests, James Grieve and Książę Albrecht Pruski were the sweetest (7.8 each). The least sweet varieties were Reneta z Brownlee (2.4), Galloway Pipping (2.5), and Szara Reneta (2.9). The Reneta varieties were the sourest, i.e., Brownlee (6.8), Szara Reneta (6.5), and Boskoop (6.3). The Książę Albrecht Pruski (1.8) and Malinowa Oberlandzka (2.0) varieties were characterised by very low perceived acidity. The varieties characterised by the lowest sweetness and sourness are gathered in Figure 2.

Apple preferences vary significantly based on ethnicity and region, with many Estonian and Northern European consumers favouring sour-sweet apples [19]. While Asian consumers predominantly prefer sweet apples, European preferences lean towards apples with a balanced firmness, juiciness, and sweetness, with about 55% of apple-eaters choosing sweet apples [19]. The studies show that European consumers are willing to choose average or lower-than-average acidity [19]. Such a balance in this study is observed in the following varieties: Koksa Pomarańczowa, Kosztela, Krótkonóżka Królewska, Piękna z Rept, and Reneta Harberta. People with diabetes are advised to select apple varieties with lower sweetness and reduced acidity. Apples characterised by low sugar content but high acidity are generally not palatable. The acceptability of low sweetness is contingent upon the absence of compensatory high acidity. Hence, preferable varieties are Galloway Pippin, Niezrównane Peasgooda, and Pepina Lineusza.

### 3.3. Acidity

Table 3 displays the mean acidity values for each variety analysed and the standard deviation to indicate variability within each variety.

The highest values for acidity, expressed in terms of the mass of malic acid per 100 g of fruit, were observed for the varieties Reneta Kulona and Reneta z Brownlee (1.14 each), as well as for Galloway Pippin (1.11), Boskoop (1.10), and Szara Reneta (1.09). The Kosztela variety had the lowest content of acidic compounds (0.26). Reneta apples, known for acidity [20], showed relatively high acidity. All varieties of Reneta apples had sourness levels higher than the average (from 0.70 in the case of Reneta Blenheim to 1.14 for Reneta z Brownlee and Kulona). Statistical analysis showed a clear correlation between the sensory assessment of apple sourness and measured acidity. The Pearson’s r correlation coefficient for these two sets was 0.60, suggesting a strong positive correlation. A similar statistical analysis was also carried out; however, considering the influence of perceived sweetness, the Pearson’s r correlation coefficient was as high as 0.76. That shows a clear tendency for consumers to perceive much lower sourness in the case of apple fruit with high sweetness.

Sensory evaluation was necessary to emphasise which kind of apple consumers are inclined to choose the preferable balance between sourness and sweetness of apples. The sensory evaluation analysis would help distinguish the optimal variety for an individual with a specific taste. Of all the analysed varieties, it is possible to distinguish those that are tasty and, at the same time, have valuable properties for consumers, i.e., low sugar or allergen content.

### 3.4. Sugar Content

In this study, gas chromatography coupled with mass spectrometry was applied. The results are given in g/100 g of apple pulp and presented as the average for each variety (Table 4).

As indicated in Table 4, the varieties with the highest total sugar contents were Reneta Kulona (17.56 g/100 g of apple pulp), Shieblers Taubenapfel (14.68 g/100 g), and Boskoop (13.57 g/100 g). The lowest values were observed for Jakub Lebel (9.06 g/100 g), Galloway Pippin (9.71 g/100 g), and Krótkonóżka Królewska (10.02 g/100 g). In terms of total sugar content (ranging from 9.06 to 10.02 g/100 g), the best varieties for people with diabetes are Jakub Lebel, Galloway Pippin, and Krótkonóżka Królewska.

Sorbitol had the most significant impact on perceived sweetness, with the highest correlation coefficient of 0.78. Almost no correlation was observed for sucrose (the correlation coefficient was very low: 0.007). The correlation coefficients for the remaining sugars were within the range of 0.30–0.34, which indicates a weak positive correlation.

### 3.5. Polyphenol and Antioxidant Potential

Table 5 shows the average results for the analysed apple varieties’ total polyphenol content and antioxidant potential. The high antioxidant potential is indicated by an equivalent amount of Trolox measured at 4 mM Trolox Equivalents (TE). As many as 13 varieties exceeded this value. The highest values were measured for the Książę Albrecht Pruski, Krótkonóżka Królewska, and Grochówka varieties (5.70, 5.68, and 5.50 mM TE, respectively). Kosztela and Grafsztynek Inflancki were characterised by relatively low antioxidant potential (1.64 and 1.92 mM TE, respectively). The highest total polyphenol contents were observed in the varieties James Grieve (63.3 mg GAE/100 g of fruit), Złota Renata (57.6 mg GAE/100 g of fruit), and Schieblers Taubenapfel (53.0 mg GAE/100 g of fruit). The Pepina Ribstona variety had the lowest polyphenol content, at only 28.6 mg GAE/100 g of fruit, followed by Galloway Pippin (28.9 mg GAE/100 g) and Pepina Linneusza (29.8 mg GAE/100 g). A correlation was observed between the antioxidant potential and total polyphenol content. The Pearson’s r correlation coefficient was 0.67, which indicates a clear positive correlation. Values for total polyphenol content were expressed as the equivalent amount of gallic acid [mg GAE/100 g of fresh weight (FW)]. The results are presented as the means for each sample ± the standard deviation.

By comparing the sugar content, perceived sweetness, antioxidant potential, and polyphenol content, the most suitable apple varieties for people with diabetes can be identified, providing a high dose of antioxidants and polyphenols. The Jakub Lebel variety has a relatively low sugar content (9.06 g/100 g) and high levels of polyphenols and antioxidants (41.1 ± 0.20 and 4.51 ± 0.52, respectively). A similar correlation was observed in Krótkonóżka Królewska, with 10.2 g/100 g of total sugar content, 4.33 ± 0.29 mM TE/g of DPPH antioxidant potential, and 46.2 ± 0.15 mg GAE/100 g total polyphenol content. The Galloway Pippin variety was found to have a low sugar content of 9.71 g/100 g, corresponding to a perceived sweetness of 2.5. Książę Albert Pruski showed a high DPPH antioxidant potential (4.54 ± 0.44 mM TE/g) with a low sugar content (11.09 mM TE/g). The James Grieve apple variety provides a high polyphenol content of 63.3 ± 0.11 with a high antioxidant level of 4.36 ± 0.47 mM TE/g. Grochówka offers a high polyphenol content of 48.6 ± 0.19 mg GAE/100 g, a high level of antioxidant protection at 4.42 ± 0.18 mM TE/g, and a low level of perceived sweetness at 3.0. The Pearson’s r correlations for individual varieties were as follows: The correlation between DPPH and FRAP was 0.98, indicating a strong logical and consistent relationship between the two methods used to measure antioxidant potential. The correlation between TPC and DPPH was 0.60, highlighting a clear relationship between polyphenol content and antioxidant potential. The correlation between TPC and FRAP was 0.63. These results confirm the consistent and significant relationship between the tested samples’ polyphenol content and antioxidant potential.

Based on the obtained results, Koksa Pomarańczowa and Książę Albrecht Pruski were identified as varieties the most suitable for people with allergies since they are characterised by the lowest content of tested allergens. For people with diabetes, the most suitable apple variety was found to be Jakub Lebel, providing large doses of antioxidants and polyphenols.

## 4. Discussion

Apples are popular fruits renowned for their nutritional value and positive impact on health. The presence of phenolic compounds in apples reduces the risk of cardiovascular diseases and type 2 diabetes [11]. These polyphenols exhibit anti-inflammatory effects that are beneficial for allergic reactions and can diminish the allergenicity of various food sources, including apples [21]. This study aimed to identify apple varieties suitable for allergy sufferers and individuals with diabetes.

The composition of soluble sugars such as fructose, sucrose, glucose, and also sorbitol significantly influences fresh fruit’s quality and commercial value [22]. In 2023, Shuhui Zhang and coworkers [23] investigated the regulation of the sugar transporter gene MdSWEET9b, which plays a pivotal role in sugar accumulation during apple development. Their findings revealed a strong correlation between sucrose and total sugar content, with sucrose holding the highest correlation coefficient at 0.867—indicating an exceptionally significant positive correlation.

The correlation analyses conducted in this study highlight the impact of different sugar components on perceived sweetness. Surprisingly, sorbitol emerged as the most influential factor, showing the highest correlation coefficient of 0.43. Sucrose exhibited practically no correlation with a coefficient close to zero (0.007). The remaining sugars analysed displayed weak positive correlations, with correlation coefficients ranging from 0.30 to 0.34. Consequently, the order of influence was as follows: sorbitol > glucose > fructose > sucrose. These insights contribute to a better understanding of the perception of sweetness in apples, providing valuable information for consumers and the fruit industry.

The main challenge with assessing the sugar content in apples is that these compounds change dynamically in quantity as the fruit ripens. Moreover, fruit on trees ripens unevenly, depending mainly on the sun and shade it is exposed to, the rainfall intensity, and average air temperature. All this makes it virtually impossible to collect apple fruit samples at the same level of ripeness for all seasons, varieties, trees, et cetera. Therefore, analysis of the results of assessments, even at the time of collection, will be subject to some errors [24]. The impact of these factors can be reduced by increasing the number of samples tested and the number of analyses performed. There are three main sugars present in apples, i.e., fructose, glucose, and sucrose. Their quantitative determination is possible using liquid chromatography (LC) as well as gas chromatography (GC) [24]. Similar results, reflecting a consistent total sugar content, were reported by Ticha and coworkers [24] in 2015, although different apple varieties were studied. Their study focused on the sugar composition of various apple varieties in apple homogenate and the correlation with sensory evaluation. They aimed to identify apple varieties suitable for individuals with obesity and diabetes. For the 17 studied varieties of apple, Ticha and coworkers reported a range of total sugars from 10.1 (Selena, Ontario) to 16.1 (Boskoop) grammes per 100 g of apple. In our study, the total sugar content in the tested varieties varied from 9.06 (Jakub Label) to 14.68 (Schieblers Taubenapfel) grammes per 100 g of apples. The perceived sweetness in the study by Ticha and coworkers ranged from 12.2 (Selena) to 19.5 (Rajka, Boskoop). Our results ranged from 2.4 (Reneta with Brownlee) to 7.8 (Prince Albert of Prussia, James Grieve). The varieties recommended by Ticha and coworkers for diabetic patients were Selena and Ontario. The slight variations in the sweetness results may be explained by the subjective nature of sensory verification conducted by individuals with varied perceptions of sweetness.

The Jakub Lebel variety exhibited the lowest sugar content (9.06 g/100 g), while the least sweet varieties were Reneta z Brownlee (2.4) and Galloway Pippin (2.5). The varieties with the lowest acidic compound content were Książę Albrecht Pruski (1.8), Malinowa Oberlandzka, James Grieve (2.0 each), Reneta Kulona (3.0), and Kantówka Gdańska (3.3). These findings provide valuable insights for individuals managing diabetes, enabling them to make informed choices about apple varieties with lower sugar levels.

Significant amounts of both Bet v 1 homologues and profilins were found in the studied apple varieties. Kosztela exhibited the lowest amounts of Bet v 1 homologues (4.21 ± 0.01 µg/g), followed by Koksa Pomarańczowa (4.24 ± 0.08 µg/g), Kantówka Gdańska (4.68 ± 0.59 µg/g), and Reneta z Brownlee (4.86 ± 0.18 µg/g). Reneta Harberta demonstrated the lowest level of profilins (1.74 ± 0.22 ng/g), closely followed by Schieblers Taubenapfel (2.26 ± 0.12 ng/g). Given their minimal content of both profilins and Bet v 1 homologues, Koksa Pomarańczowa (4.24 ± 0.08 µg/g Bet v 1 and 4.49 ± 0.82 ng/g profilins) and Książę Albrecht Pruski (5.57 ± 0.07 µg/g Bet v 1 and 3.34 ± 0.09 ng/g profilins) emerged as two varieties with noteworthy values for people with allergies.

In 2021, Siekierzyńska and coworkers [25] employed molecular and immunological methods to identify apple varieties with low allergen content. The analysed samples included other varieties investigated in the present study, such as Kosztela, Grochówka, Jakub Lebel, and Kantówka Gdańska. The results in the two studies exhibit some similarities—the same varieties were characterised by lower allergen content. For instance, Siekierzyńska and coworkers reported the Mal d 1 allergen content as 0.3 µg/g in Kantówka Gdańska, 0.6 µg/g in Kosztela, 9.2 µg/g in Grochówka, and 17.5 µg/g in Jakub Lebel. In our publication, the respective results were 4.68 µg/g, 4.21 µg/g, 5.45 µg/g, and 8.87 µg/g. Discrepancies can be attributed to various factors, including cultivation methods, soil conditions, storage methods, and duration [7]. Hallmann et al. in 2020 [26] found a strong correlation between Bet v 1 homologues and anthocyanins in raspberries. However, no similar correlations between individual allergens and polyphenols, or their total values, were identified in our work. In raspberries, the polyphenols are distributed evenly throughout the fruit, much like the allergens. In contrast, in apples, the allergens are predominantly present in the pulp (juice), while polyphenols are primarily concentrated in the peel. The polyphenol content in apple pulp is relatively low. The absence of a positive correlation between allergens and polyphenols is encouraging, as it provides the opportunity to identify apple varieties with low allergen content and simultaneously high levels of health-promoting compounds [27].

Scientific research supports the notion that apple polyphenols can help alleviate allergic rhinitis, commonly known as hay fever. In a study conducted by Enomoto [28], which investigated the clinical effects of apple polyphenols on chronic allergic rhinitis through a randomised, double-blind, placebo-controlled study involving 33 individuals with chronic allergic rhinitis, a notable improvement was observed in the frequency of sneezing attacks and nasal discharge following the administration of a small or large dose of apple polyphenol extract [28]. These findings suggest that apple polyphenols can relieve hay fever sufferers, offering an alternative to traditional allergy medications.

The apple varieties exhibited high antioxidant potential, determined by the DPPH method, and high total polyphenol content, determined by the Folin–Ciocalteu method. Remarkably, 13 varieties showed a relatively high antioxidant potential value, surpassing the equivalent amount of Trolox at 4 mM TE. Krośniak [29] and coworkers reported significantly different results, possibly influenced by study methodologies and the sample type (apple juice rather than pulp extracts). Nonetheless, the results for many varieties were comparable. Piękna z Rept, Koksa Pomarańczowa, Kronselska, and Kosztela cultivars exhibited lower antioxidant potential content (4.2, 4.2, 5.1, and 5.1 mM TE/g, respectively). The results for these cultivars in this study were even lower, at 3.22, 2.55, 2.14, and 1.64 mM TE/g, respectively. The varieties Reneta Kulona (7.1 mM TE/g) and Złota Reneta (7.9 mM TE/g) had relatively high values, which is in accordance with the results of our study (4.30 and 4.02 mM TE/g).

In 2011, Li Fu and coworkers [30] explored the antioxidant capacity and total phenolic contents of 62 fruits, including apples. Their results, though comparable to ours, revealed lower total polyphenol content in the tested apples (ranging from 58.12 ± 3.98 to 73.96 ± 3.52 mg GAE/100 g). A broader range of polyphenol contents may be observed, from 137.4 ± 9.0 (Malinowa Oberlandzka) to 246.6 ± 12.4 mg GAE/100 g (James Grieve), almost tripling the polyphenol content in the analysed fruits. The values for antioxidant potential reported by Li Fu and coworkers were in the range of 4.25 ± 0.27 to 5.47 ± 0.04 μmol TE/g. In our study, presented in mM Trolox/100 g of fruit, the content ranged from 1.64 ± 0.41 (Kosztela) to 4.54 ± 0.44 (Książe Albrecht Pruski). The discrepancies in antioxidant potential may be attributed to differences in the study methodologies. Notably, Li Fu’s research analysed apple pressed juice, not peel extracts, potentially accounting for differences in Trolox content per 100 g of raw material. Both our results and those of Li Fu’s team provide valuable insights for consumers, dietitians, and food policymakers regarding the antioxidant function of apple fruit.

Based on the results of our study, the apple fruit varieties ranked from lowest to highest Bet v I homologue content (4.21–5.77 µg/g) were as follows: Kosztela, Koksa Pomarańczowa, Kantówka Gdańska, Reneta z Brownlee, Grochówka, Książe Albert Pruski, Reneta Blenheimska, and Kalwila Aderslebeńska. For profilin (1.74–3.98 ng/g), Reneta Harberta, Schieblers Taubenapfel, Deans’ Codlin, Kronselska, Książę Albrecht Pruski, and Kalwila Aderslebeńska.

The varieties that exhibited the lowest total sugar content (9.71–10.02 g/100 g of raw material) were Jakub Lebel, Galloway Pippin, and Krótkonóżka Królewska. These apples are the most suitable varieties for individuals with diabetes. The James Grieve variety is an excellent choice for individuals aiming to maximise antioxidant or polyphenol intake, providing a high polyphenol content of 63.3 ± 0.11 coupled with a robust antioxidant level of 5.34 ± 0.45. For those preferring low perceived sweetness (3.0), the optimal option would be Grochówka, offering 48.6 ± 0.19 in polyphenol content along with a substantial antioxidant protection level of 5.50 ± 0.34. The Jakub Lebel variety, with a relatively low sugar content (9.06), boasts a high polyphenol and high antioxidant content, at 41.1 ± 0.20 and 5.16 ± 0.42, respectively. Another noteworthy variety is Krótkonóżka Królewska, with a sugar content of 10.2, accompanied by antioxidant levels of 5.68 ± 0.49 and polyphenols at 46.2 ± 0.15. Galloway Pippin, with a low sugar content of 9.71 and a perceived sweetness of 2.5, also stands out. Książę Albrecht Pruski combines high antioxidant potential (5.70 ± 0.77) with a low sugar content of 11.09.

**Figure 3:**
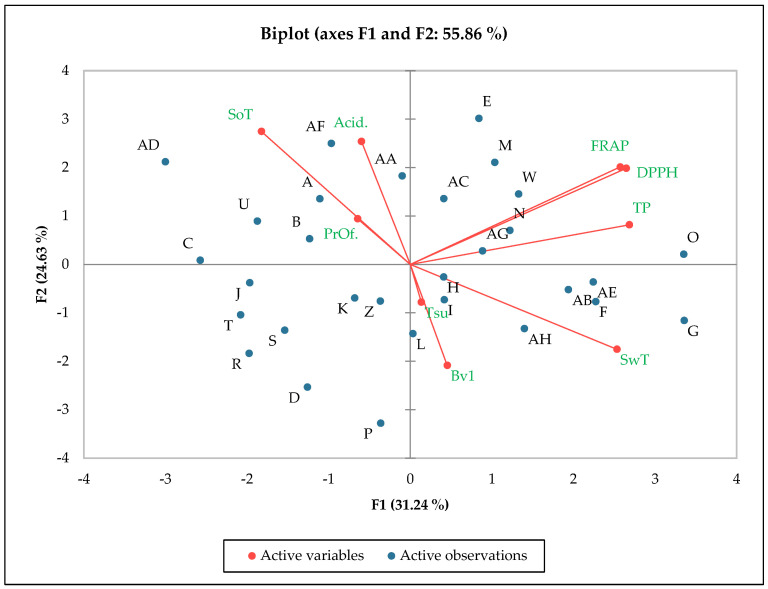
PCA analysis showing the relationship between the chemical composition and chemical composition of different apple cultivars. (PrOf.) Profilin, (Acid.) Acidity, (TP) Total polyphenol, (DPPH) Antioxidant potential DPPH, (FRAP) Antioxidant potential FRAP, (SwT) Sweet test, (SoT) Sour test, and (Tsu) Total sugars. Experimental objects: Boskoop (A), Deans’ Codlin (B), Galloway Pippin (C), Grafsztynek Inflancki (D), Grochówka (E), Jakub Lebel (F), James Grieve (G), Kalwila Aderslebeńska (H), Kantówka Gdańska (I), Koksa Pomarańczowa (J), Kosztela (K), Kronselska (L), Krótkonóżka Królewska (M), Książe Albert (N), Książe Albrecht Pruski (O), Malinowa Oberlandzka (P) Niezrównane Peasgooda (R), Pepina Lineusza (S), Pepina Ribstona (T), Piękna z Rept (U), Reneta Blenheimska (W), Reneta Harberta (Z), Reneta Kanadyjska (AA), Reneta Kulona (AB), Reneta Strauwalda (AC), Reneta z Brownlee (AD), Schieblers Taubenapfel (AE), Szara Reneta (AF), Złota Reneta (AG), and Złotka Kwidzyńska (AH).

Based on the PCA results, the overall degree of variability explained by F1 and F2 was 55.86% for examined apple cultivars and chemical analysis of apples. This result was confirmed by a strong link between the chemical composition of domestic and old apple cultivars and measured features such as antioxidant activity, total polyphenol content, sensory features, and anti-allergy compounds. All examined apple cultivars can be divided into different groups. Six cultivars would belong to the first group, i.e., Boskoop, Deans’ Codlin, Galloway Pipping, Piękna z Rept, Reneta Kanadyjska, and Szara Reneta. These are strongly linked with sensory determinants such as sourness and acidity measured by the chemical method. What is more, a strong relationship between profilin content is observed. A second group of relationships can be observed between antioxidant activity (measured by the FRAP and DPPH methods and total polyphenol content) and the following apple cultivars: Grochowka, Kalwila Aderslebeńska, Książę Albert, Książę Albert Pruski, Reneta Strauwalda, and Złota Reneta. It is worth noticing that these cultivars were located in a separate chart area next to the previously described right. Apple cultivars such as Jacub Lebel, James Grieve, Kalwila Aderslebeńska, Kantówka Gdanska, Kosztela, Reneta Kulona, Schieblers Taubenapfel, and Złotka Kwidzyńska showed a strong link with sweet sensory taste, Bet v 1 content, and total sugar content. The last apple cultivar group was created from apples Grafsztyn, Kosztela, Malinowa Oberlandzka, Niezrównane Peasgooda, Pepina Linnaeusza, Pepina Ribstona, and Reneta Herberta, which do not show any interactions on a significant level (Figure 3).

## 5. Conclusions

This analysis provides valuable guidance for individuals managing diabetes and allergies, helping them choose apple varieties that align with their dietary needs and health considerations. For individuals dealing with health challenges, closely monitoring levels of potentially unfavourable ingredients and staying well-informed about products that meet their unique dietary needs is crucial.

Given their minimal content of both profilins and Bet v 1 homologues, Koksa Pomarańczowa (4.24 ± 0.08 µg/g Bet v 1 and 4.49 ± 0.82 ng/g profilins) and Książę Albrecht Pruski (5.57 ± 0.07 µg/g Bet v 1 and 3.34 ± 0.09 ng/g profilins) were identified as suitable for people with allergies. For people with diabetes, the most suitable apple variety was found to be Jakub Lebel, providing large doses of antioxidants and polyphenols (41.1 ± 0.20 mg GAE/100 g and 5.16 ± 0.42 mM TE/g, respectively) and a relatively low sugar content (9.06 mg/100 g).

## Figures and Tables

**Table 1:** Allergen content (Bet v 1 homologs and profilins) in apple juices. All analysed varieties were gathered in the 2019 season. The results are presented as mean ± standard deviation. Statistical significance in Supplementary Tables S1 and S2.

Variety	Content of Bet v 1 Homologs [µg/g]	Content of Profilins [ng/g]
Boskoop	6.45 ± 0.09	3.41 ± 0.03
Deans’ Codlin	6.48 ± 0.08	2.90 ± 0.03
Galloway Pipping	6.97 ± 0.03	4.33 ± 0.02
Grafsztynek Inflancki	6.85 ± 0.04	4.94 ± 0.05
Grochówka	5.45 ± 0.09	5.40 ± 0.03
Jakub Lebel	8.87 ± 0.01	5.05 ± 0.09
James Grieve	8.05 ± 0.07	5.95 ± 0.07
Kalwila Aderslebeńska	5.77 ± 0.09	3.98 ± 0.08
Kantówka Gdańska	4.68 ± 0.09	5.71 ± 0.06
Koksa Pomarańczowa	4.24 ± 0.08	4.49 ± 0.02
Kosztela	4.21 ± 0.01	4.28 ± 0.07
Kronselska	6.93 ± 0.05	3.16 ± 0.01
Krótkonóżka Królewska	6.10 ± 0.06	6.15 ± 0.03
Książę Albert	6.97 ± 0.02	7.26 ± 0.05
Książę Albrecht Pruski	5.57 ± 0.07	3.34 ± 0.09
Malinowa Oberlandzka	9.86 ± 0.07	7.37 ± 0.09
Niezrównane Peasgooda	8.09 ± 0.08	6.49 ± 0.02
Pepina Linneusza	7.34 ± 0.01	7.21 ± 0.02
Pepina Ribstona	6.97 ± 0.03	7.67 ± 0.07
Piękna z Rept	6.38 ± 0.07	5.73 ± 0.06
Reneta Blenheimska	5.73 ± 0.01	8.81 ± 0.05
Reneta Harberta	7.47 ± 0.04	1.74 ± 0.02
Reneta Kanadyjska	6.81 ± 0.04	7.65 ± 0.03
Reneta Kulona	7.59 ± 0.82	3.66 ± 0.04
Reneta Strauwalda	6.50 ± 0.02	6.74 ± 0.03
Reneta z Brownlee	4.86 ± 0.08	7.63 ± 0.09
Schieblers Taubenapfel	6.52 ± 0.03	2.26 ± 0.02
Szara Reneta	6.76 ± 0.09	7.88 ± 0.07
Złota Reneta	6.81 ± 0.05	7.64 ± 0.04
Złotka Kwidzyńska	12.40 ± 0.07	3.87 ± 0.02

**Table 2:** Results of sensory evaluation. Statistical significance in Supplementary Tables S3 and S4.

Variety	Sweet	Sour
Boskoop	3.3 ± 0.4	6.3 ± 0.2
Deans’ Codlin	3.3 ± 0.3	5.5 ± 0.3
Galloway Pippin	2.5 ± 0.2	4.3 ± 0.1
Grafsztynek Inflancki	6.5 ± 0.6	3.5 ± 0.3
Grochówka	3.0 ± 0.2	5.3 ± 0.2
Jakub Lebel	7.4 ± 0.3	3.4 ± 0.3
James Grieve	7.8 ± 0.4	2.0 ± 0.4
Kalwila Aderslebeńska	7.5 ± 0.9	3.5 ± 0.5
Kantówka Gdańska	6.1 ± 0.4	3.3 ± 0.2
Koksa Pomarańczowa	5.4 ± 0.6	5.7 ± 0.3
Kosztela	6.3 ± 0.2	4.5 ± 0.2
Kronselska	6.5 ± 0.3	3.5 ± 0.1
Krótkonóżka Królewska	5.3 ± 0.5	4.7 ± 0.2
Książę Albert	6.6 ± 0.4	4.2 ± 0.1
Książę Albrecht Pruski	7.8 ± 0.1	1.8 ± 0.2
Malinowa Oberlandzka	7.4 ± 0.1	2.0 ± 0.0
Niezrównane Peasgooda	3.0 ± 0.5	3.5 ± 0.1
Pepina Linneusza	4.3 ± 0.2	3.7 ± 0.2
Pepina Ribstona	4.5 ± 0.4	4.0 ± 0.0
Piękna z Rept	4.7 ± 0.6	5.3 ± 0.2
Reneta Blenheimska	7.3 ± 0.5	4.8 ± 0.3
Reneta Harberta	5.3 ± 0.5	4.5 ± 0.4
Reneta Kanadyjska	4.3 ± 0.4	5.3 ± 0.3
Reneta Kulona	7.3 ± 0.6	3.0 ± 0.2
Reneta Strauwalda	4.7 ± 0.7	5.0 ± 0.0
Reneta z Brownlee	2.4 ± 0.2	6.8 ± 0.2
Schieblers Taubenapfel	7.3 ± 0.3	3.5 ± 0.1
Szara Reneta	2.9 ± 0.2	6.5 ± 0.3
Złota Reneta	6.3 ± 0.4	4.8 ± 0.2
Złotka Kwidzyńska	6.5 ± 0.3	3.5 ± 0.5

**Table 3:** Average acidity of analysed apple varieties. Statistical significance in Supplementary Table S5.

Variety	Acidity [g Malic Acid Equivalent/100 g Apple]
Boskoop	1.10 ± 0.04
Deans’ Codlin	0.75 ± 0.01
Galloway Pipping	1.11 ± 0.02
Grafsztynek Inflancki	0.41 ± 0.03
Grochówka	0.84 ± 0.08
Jakub Lebel	0.52 ± 0.09
James Grieve	0.40 ± 0.03
Kalwila Aderslebeńska	0.95 ± 0.03
Kantówka Gdańska	0.45 ± 0.02
Koksa Pomarańczowa	0.58 ± 0.02
Kosztela	0.26 ± 0.02
Kronselska	0.78 ± 0.02
Krótkonóżka Królewska	0.80 ± 0.03
Książę Albert	0.82 ± 0.01
Książę Albrecht Pruski	0.76 ± 0.01
Malinowa Oberlandzka	0.42 ± 0.01
Niezrównane Peasgooda	0.68 ± 0.02
Pepina Linneusza	0.39 ± 0.02
Pepina Ribstona	0.72 ± 0.28
Piękna z Rept	0.92 ± 0.02
Reneta Blenheimska	0.70 ± 0.01
Reneta Harberta	0.71 ± 0.02
Reneta Kanadyjska	0.98 ± 0.03
Reneta Kulona	1.14 ± 0.29
Reneta Strauwalda	0.79 ± 0.05
Reneta z Brownlee	1.14 ± 0.00
Schieblers Taubenapfel	0.78 ± 0.02
Szara Reneta	1.09 ± 0.22
Złota Reneta	0.51 ± 0.06
Złotka Kwidzyńska	0.53 ± 0.01

**Table 4:** Average sugar and sorbitol content for all analysed varieties. Statistical significance in Supplementary Tables S6–S11.

Variety	Fructose	Glucose	Xylose	Sucrose	Sorbitol	Total Sugars [g/100 g of Apple Pulp]
Boskoop	7.83 ± 0.51	1.80 ± 0.29	0.00 ± 0.00	3.94 ± 0.33	0.21 ± 0.01	13.57 ± 0.54
Deans’ Codlin	6.70 ± 0.43	2.02 ± 0.27	0.01 ± 0.00	4.27 ± 0.27	0.44 ± 0.04	13.00 ± 0.45
Galloway Pippin	5.41 ± 0.32	0.98 ± 0.23	0.02 ± 0.01	3.30 ± 0.29	0.30 ± 0.01	9.71 ± 0.37
Grafsztynek Inflancki	5.96 ± 0.23	1.32 ± 0.13	0.04 ± 0.02	4.05 ± 0.31	0.67 ± 0.07	11.37 ± 0.43
Grochówka	5.49 ± 0.17	1.22 ± 0.14	0.03 ± 0.01	3.93 ± 0.30	0.41 ± 0.04	10.66 ± 0.39
Jakub Lebel	4.81 ± 0.30	1.57 ± 0.21	0.00 ± 0.00	2.68 ± 0.26	0.62 ± 0.10	9.06 ± 0.36
James Grieve	6.33 ± 0.22	1.21 ± 0.10	0.08 ± 0.02	4.15 ± 0.29	0.78 ± 0.11	11.77 ± 0.45
Kalwila Aderslebeoska	5.96 ± 0.33	1.34 ± 0.12	0.10 ± 0.04	4.64 ± 0.34	0.59 ± 0.07	12.04 ± 0.40
Kantówka Gdańska	6.26 ± 0.45	1.20 ± 0.17	0.10 ± 0.04	3.98 ± 0.27	0.55 ± 0.12	11.53 ± 0.38
Koksa Pomarańczowa	7.67 ± 0.48	1.62 ± 0.18	0.04 ± 0.03	3.23 ± 0.25	0.60 ± 0.10	12.56 ± 0.46
Kosztela	6.03 ± 0.27	1.12 ± 0.11	0.07 ± 0.03	4.13 ± 0.23	0.54 ± 0.07	11.34 ± 0.44
Kronselska	5.65 ± 0.24	1.61 ± 0.20	0.08 ± 0.03	3.57 ± 0.30	0.62 ± 0.06	10.91 ± 0.41
Krótkonóżka Królewska	5.90 ± 0.40	1.05 ± 0.09	0.02 ± 0.00	3.06 ± 0.27	0.48 ± 0.06	10.02 ± 0.33
Książę Albert	4.86 ± 0.18	1.23 ± 0.15	0.12 ± 0.03	4.76 ± 0.32	0.39 ± 0.05	10.97 ± 0.41
Książę Albrecht Pruski	5.11 ± 0.26	1.06 ± 0.10	0.04 ± 0.04	4.88 ± 0.22	0.74 ± 0.14	11.09 ± 0.44
Malinowa Oberlandzka	6.97 ± 0.31	1.32 ± 0.31	0.04 ± 0.03	4.38 ± 0.31	0.66 ± 0.11	12.70 ± 0.53
Niezrównane Peasgooda	6.28 ± 0.34	1.16 ± 0.27	0.00 ± 0.00	5.37 ± 0.39	0.42 ± 0.04	12.81 ± 0.55
Pepina Linneusza	5.74 ± 0.27	1.32 ± 0.19	0.07 ± 0.04	3.16 ± 0.17	0.51 ± 0.07	10.28 ± 0.24
Pepina Ribstona	5.08 ± 0.29	0.77 ± 0.07	0.00 ± 0.00	6.91 ± 0.42	0.42 ± 0.01	12.76 ± 0.43
Piękna z Rept	5.34 ± 0.31	1.02 ± 0.11	0.07 ± 0.04	4.31 ± 0.35	0.42 ± 0.04	10.74 ± 0.35
Reneta Blenheimska	5.02 ± 0.20	0.84 ± 0.09	0.00 ± 0.00	6.68 ± 0.31	0.58 ± 0.11	12.54 ± 0.47
Reneta Harberta	5.83 ± 0.28	1.07 ± 0.12	0.01 ± 0.00	4.40 ± 0.28	0.54 ± 0.07	11.31 ± 0.38
Reneta Kanadyjska	5.17 ± 0.31	0.86 ± 0.14	0.00 ± 0.00	5.16 ± 0.29	0.40 ± 0.03	11.19 ± 0.36
Reneta Kulona	8.46 ± 0.53	2.90 ± 0.28	0.00 ± 0.00	6.20 ± 0.33	0.51 ± 0.07	17.56 ± 0.56
Reneta Strauwalda	5.42 ± 0.43	1.36 ± 0.33	0.11 ± 0.04	5.45 ± 0.35	0.37 ± 0.01	12.34 ± 0.49
Reneta z Brownlee	4.79 ± 0.23	0.98 ± 0.16	0.00 ± 0.00	4.63 ± 0.28	0.41 ± 0.01	10.40 ± 0.36
Schieblers Taubenapfel	5.87 ± 0.38	1.51 ± 0.26	0.02 ± 0.01	7.28 ± 0.38	0.72 ± 0.14	14.68 ± 0.42
Szara Reneta	5.09 ± 0.33	1.37 ± 0.29	0.01 ± 0.00	5.05 ± 0.36	0.77 ± 0.15	11.52 ± 0.39
Złota Reneta	5.21 ± 0.32	1.23 ± 0.20	0.02 ± 0.00	6.26 ± 0.27	0.74 ± 0.14	12.72 ± 0.50
Złotka Kwidzyńska	5.37 ± 0.41	1.09 ± 0.21	0.10 ± 0.04	4.28 ± 0.26	0.65 ± 0.08	10.84 ± 0.46

**Table 5:** The total polyphenol content of all analysed varieties correlated with antioxidant potential. Statistical significance in Supplementary Tables S12–S14.

Variety	Total Polyphenol Content [mg GAE/100 g of FW]	Antioxidant Potential DPPH [mM TE/g]	Antioxidant Potential FRAP [mM TE/g]
Boskoop	44.1 ± 0.27	3.40 ± 0.45	3.27 ± 0.55
Deans’ Codlin	38.6 ± 0.12	3.33 ± 0.47	3.45 ± 0.67
Galloway Pipping	28.9 ± 0.21	2.86 ± 0.48	2.23 ± 0.15
Grafsztynek Inflancki	33.7 ± 0.14	1.92 ± 0.32	1.05 ± 0.27
Grochówka	48.6 ± 0.19	4.42 ± 0.18	5.50 ± 0.34
Jakub Lebel	41.1 ± 0.20	4.51 ± 0.52	5.16 ± 0.42
James Grieve	63.3 ± 0.11	4.36 ± 0.47	5.34 ± 0.45
Kalwila Aderslebeńska	32.3 ± 0.10	3.99 ± 0.43	4.44 ± 0.56
Kantówka Gdańska	42.6 ± 0.23	3.80 ± 1.32	4.02 ± 0.29
Koksa Pomarańczowa	38.9 ± 0.11	2.55 ± 0.34	1.98 ± 0.13
Kosztela	33.8 ± 0.90	1.64 ± 0.41	1.09 ± 0.22
Kronselska	46.6 ± 0.13	2.14 ± 0.22	1.99 ± 0.31
Krótkonóżka Królewska	46.2 ± 0.15	4.33 ± 0.29	5.68 ± 0.49
Książę Albert	42.8 ± 0.70	4.23 ± 0.70	5.32 ± 0.67
Książę Albrecht Pruski	51.3 ± 0.27	4.54 ± 0.44	5.70 ± 0.77
Malinowa Oberlandzka	33.6 ± 0.10	2.45 ± 0.25	2.25 ± 0.38
Niezrównane Peasgooda	31.9 ± 0.80	2.58 ± 0.36	2.17 ± 0.24
Pepina Linneusza	29.8 ± 0.10	3.06 ± 0.18	3.05 ± 0.35
Pepina Ribstona	28.6 ± 0.90	2.83 ± 0.28	2.50 ± 0.32
Piękna z Rept	30.2 ± 0.17	3.22 ± 0.16	3.27 ± 0.20
Reneta Blenheimska	42.7 ± 0.12	4.63 ± 0.37	4.96 ± 0.56
Reneta Harberta	40.0 ± 0.15	3.24 ± 0.25	3.31 ± 0.54
Reneta Kanadyjska	50.0 ± 0.18	4.01 ± 0.14	4.52 ± 0.68
Reneta Kulona	47.0 ± 0.13	4.30 ± 0.37	4.49 ± 0.43
Reneta Strauwalda	40.1 ± 0.16	4.42 ± 0.45	4.94 ± 0.75
Reneta z Brownlee	33.0 ± 0.90	3.00 ± 0.27	2.53 ± 0.29
Schieblers Taubenapfel	53.0 ± 0.50	4.30 ± 0.38	4.80 ± 0.57
Szara Reneta	39.8 ± 0.89	4.24 ± 0.32	4.81 ± 0.61
Złota Reneta	57.6 ± 0.10	4.02 ± 0.18	4.44 ± 0.39
Złotka Kwidzyńska	51.8 ± 0.20	3.96 ± 0.53	4.06 ± 0.42

## Data Availability

The original contributions presented in the study are included in the article; further inquiries can be directed to the corresponding author(s).

## Supplementary Materials

The following supporting information can be downloaded at: https://www.mdpi.com/article/10.3390/nu16132109/s1. Table S1: Summary of statistical significance results for Bet v 1 homologs. Green indicates *p* < 0.05, red indicates *p* > 0.05. Table S2: Summary of statistical significance results for profilins. Green indicates *p* < 0.05, red indicates *p* > 0.05. Table S3: Summary of statistical significance results for sensory evaluation—sour. Green indicates *p* < 0.05, red indicates *p* > 0.05. Table S4: Summary of statistical significance results for sensory evaluation—sweet. Green indicates *p* < 0.05, red indicates *p* > 0.05. Table S5: Summary of statistical significance results for acidity. Green indicates *p* < 0.05, red indicates *p* > 0.05. Table S6: Summary of statistical significance results for fructose. Green indicates *p* < 0.05, red indicates *p* > 0.05. Table S7: Summary of statistical significance results for glucose. Green indicates *p* < 0.05, red indicates *p* > 0.05. Table S8: Summary of statistical significance results for xylose. Green indicates *p* < 0.05, red indicates *p* > 0.05. Table S9: Summary of statistical significance results for sucrose. Green indicates *p* < 0.05, red indicates *p* > 0.05. Table S10: Summary of statistical significance results for sorbitol. Green indicates *p* < 0.05, red indicates *p* > 0.05. Table S11: Summary of statistical significance results for total sugar content. Green indicates *p* < 0.05, red indicates *p* > 0.05. Table S12: Summary of statistical significance results for total polyphenol content. Green indicates *p* < 0.05, red indicates *p* > 0.05. Table S13: Summary of statistical significance results for antioxidant potential DPPH. Green indicates *p* < 0.05 and red indicates *p* > 0.05. Table S14: Summary of statistical significance results for antioxidant potential FRAP. Green indicates *p* < 0.05, red indicates *p* > 0.05.
